# A Biological Study of Anisotropic Silver Nanoparticles and Their Antimicrobial Application for Topical Use

**DOI:** 10.3390/vetsci8090177

**Published:** 2021-08-31

**Authors:** Saengrawee Thammawithan, Pawinee Siritongsuk, Sawinee Nasompag, Sakda Daduang, Sompong Klaynongsruang, Nuvee Prapasarakul, Rina Patramanon

**Affiliations:** 1 Department of Biochemistry, Faculty of Science, Khon Kaen University, Khon Kaen 40002, Thailand; th_saengrawee@kkumail.com (S.T.); parbiochem@gmail.com (P.S.); somkly@kku.ac.th (S.K.); 2 Protein and Proteomics Research Center for Commercial and Industrial Purposes (ProCCI), Faculty of Science, Khon Kaen University, Khon Kaen 40002, Thailand; sakdad@kku.ac.th; 3 Genetic Engineering Interdisciplinary Program, Graduate School, Kasetsart University, Bangkok 10900, Thailand; sawinee.nas@gmail.com; 4 Division of Pharmacognosy and Toxicology, Faculty of Pharmaceutical Sciences, Khon Kaen University, Khon Kaen 40002, Thailand; 5 Department of Microbiology, Faculty of Veterinary Science, Chulalongkorn University, Bangkok 10330, Thailand; nuvee.p@chula.ac.th

**Keywords:** silver nanoparticles, anisotropic silver nanoparticles, antimicrobial resistance, alternatives to antimicrobials, *Staphylococcus pseudintermedius*

## Abstract

The excessive use of antibiotics in both human and veterinary medicine has contributed to the development and rapid spread of drug resistance in bacteria. Silver nanoparticles (AgNPs) have become a tool of choice that can be used to treat these resistant bacteria. Several studies have shown that AgNPs have antibacterial and wound healing properties. In this study, we evaluated the biological activity of anisotropic AgNPs to develop an antimicrobial gel formulation for treating wound infections. We showed that some anisotropic AgNPs (S2) have an effective antibacterial activity against bacterial pathogens and low cytotoxicity to keratinocytes and fibroblasts *in vitro*. The MIC and MBC values were in the range of 2–32 µg/mL, and cytotoxicity had IC_50_ values of 68.20 ± 9.71 µg/mL and 68.65 ± 10.97 µg/mL against human keratinocyte and normal human dermal fibroblast cells, respectively. The anisotropic AgNPs (S2) were used as a gel component and tested for antibacterial activity, including long-term protection, compared with povidone iodine, a common antiseptic agent. The results show that the anisotropic AgNPs can inhibit the growth of most tested bacterial pathogens and provide protection longer than 48 h, whereas povidone iodine only inhibits the growth of some bacteria. This study suggests that anisotropic AgNPs could be used as an alternative antimicrobial agent for treating bacterial skin infection and as a wound healing formulation.

## 1. Introduction

The frequent use of antibiotics has led to the development of bacterial resistance. There are reports of bacterial resistance to antibiotics to these species (*Escherichia coli*, *Staphylococcus aureus*, *Pseudomonas aeruginosa*, *Staphylococcus pseudintermedius*) in human and veterinary medicine [1,2,3]. Moreover, multi-drug resistant staphylococci have gradually increased worldwide [4]. In animals such as dogs, methicillin-resistant *S. pseudintermedius* (MRSP) is an issue of concern [5]. Generally, *S. pseudintermedius* is commonly found on the skin and mucosa of healthy dogs and in a small percentage of healthy cats [6,7]. Similarly to *S. aureus*, which is considered as a compound of the human normal skin flora [8], *S. pseudintermedius* is harmless in healthy individuals, but it is an opportunistic pathogen if an animal gets injured or sick [9]. With the first single observation made in 1999 from a dog in the United States, the emergence of MRSP in dogs represents a relatively new problem [10]. MRSP is a growing concern in small animal veterinary medicine. A recent study has reported that methicillin-resistant *S. pseudintermedius* strains isolated from sick dogs were 63%, and 78% of these isolates were described as being resistant to at least three antibiotic classes [11]. These “superbugs” are becoming more difficult to treat because of ineffective drugs. Therefore, discovering alternative antimicrobial agents to overcome antibiotic-resistant bacteria is important.

Silver nanoparticles (AgNPs) have attracted special attention because of their extensive properties, including good conductivity, chemical stability, unique optical and localized surface plasmon resonance [12]. Consequently, they are used in biological applications, optical imaging, data storage, sensing and as antimicrobial agents. In the last decade, AgNPs have become known as an effective antimicrobial agent with low toxicity to mammalian cells [13]. Owing to their ability to kill bacteria with complex mechanisms, they seem to have a high potential to solve the problem of multi-drug resistance bacteria [14]. Several studies have demonstrated that AgNPs have wound healing properties [15]. Therefore, AgNPs have become increasingly popular as antibiotic agents in textiles, wound dressings and medical devices. In veterinary medicine, AgNPs exhibit a strong antimicrobial effect on animal pathogens. Gurunathan et al., 2018, reported that AgNPs demonstrated significant antibacterial and anti-biofilm activities against the multidrug-resistant (MDR) pathogen of cows [16]. Moreover, AgNPs can be applied as the main antimicrobial compound or as an adjuvant to antibiotics to improve the treatment of bacterial diseases in animals [17,18]. Therefore, AgNPs could be a promising therapeutic strategy for infections caused by resistant bacteria.

AgNPs can be divided into two types according to their shape: Ag nanospheres (AgNSs) and anisotropic Ag nanoparticles (anisotropic AgNPs; have non-spherical shape such as rods, triangular nanoprisms, diamonds, octagons and thin sheets). Nowadays, AgNSs are commonly used as antimicrobial agents because of their strong antimicrobial properties and ease in synthesis. However, other AgNPs, such as anisotropic AgNPs, are also popular. Anisotropic AgNPs show potential application as chemical and biological sensors and surface-enhanced fluorescence probes, because of their powerful optical activity [19]. Moreover, anisotropic AgNPs have been reported to exhibit highly effective antimicrobial activity [20,21]. For application, anisotropic AgNPs have been used to colour wool fabrics and show high antibacterial activity against *E. coli* [22]. Some studies have demonstrated that anisotropic AgNPs have significantantibacterial activity against tested human pathogens compared with AgNSs [23]. In addition, Sukdeb Pal et al., 2007 have reported truncated triangular Ag nanoplates with a {111} plan showed the strongest biocidal action compared with spherical and rod-shaped nanoparticles [24]. In this study, we evaluated antimicrobial activity of anisotropic AgNPs against some important bacteria in both veterinary and human medicine, including their cytotoxicity to human keratinocytes and fibroblasts. Moreover, we developed an antimicrobial gel containing AgNPs as an easy way to use an anisotropic AgNP-based antimicrobial formulation for topical use, such as for infected wounds. 

## 2. Materials and Methods

### 2.1. Bacterial Strains and Culture Conditions

Five strains of *S. pseudintermedius* were obtained from the Department of Microbiology, Faculty of Veterinary Science, Chulalongkorn University. All were identified by the Vitek 2 Compact System (bioMérieux, Marcy l’Etoile, France). The oxacillin-resistant isolates (MIC ≥ 0.5 μg/mL) further underwent mecA gene existence testing using the PCR method [25]. *E. coli* O157:H7, *S. aureus* ATCC 25923 and *P. aeruginosa* ATCC 27853 were obtained from the Protein and Proteomics Research Group, Khon Kaen University. The details of these strains are shown in Table 1. Bacteria were streaked on Mueller-Hinton agar and incubated at 37 °C for 24 h. A colony was selected and inoculated with 5 mL Mueller-Hinton broth (MHB) at 37 °C overnight and sub-cultured in 5 mL of the same medium at 37 °C in a 180 rpm shaker-incubator for 3 h to yield a mid-logarithmic growth phase culture. 

### 2.2. Cell Culture

Human keratinocyte (HaCaT) cells and normal human dermal fibroblasts (NHDF) were obtained from the Protein and Proteomics Research Group, Khon Kaen University. The cells were cultured in Dulbecco’s modified Eagle’s medium with 10% heat-inactivated foetal bovine serum and 1% antibiotic: antimycotic (Gibco, Waltham, MA, USA) in a 5% CO_2_-humidified atmosphere at 37 °C. Before testing, the cells were harvested using 0.25% trypsin and seeded in 96-well plates for 24 h. 

### 2.3. Characterisation of AgNPs

AgNSs (S1) were synthesized via reduction of silver nitrate using tannic acid, and anisotropic AgNPs (S2–S5) were transformed from AgNSs by varying concentrations of hydrogen peroxide. These AgNPs were given by our collaborator Prime Nanotechnology Co., Ltd. (Bangkok, Thailand) in a stock solution of 200 µg/mL. The samples were suspended in deionised water at a concentration of 50 µg/mL. Ultraviolet-visible (UV-Vis) spectra of AgNPs were recorded by a SpectraMax M5 microplate reader (Molecular devices, San Jose, CA, USA). The shape and dispersion of the AgNPs were confirmed using a transmission electron microscope (FEI/TECNAI G2 20, FEI Company, Hillsboro, OR, USA) operating at 200 kV. The size of the AgNPs was calculated directly from the transmission electron microscopy (TEM) image using Image J software, a Java program developed by the National Institute of Mental Health, Bethesda, MD, USA).

### 2.4. Bacterial Susceptibility Test

To investigate the susceptibility of bacteria to AgNPs and antibiotic, the minimum inhibitory concentration (MIC) and minimum bactericidal concentration (MBC) were determined by a serial dilution method in a 96-well plate. Briefly, the AgNPs were diluted in deionised water by two-fold dilution at 2–200 μg/mL. These solutions were added to a 96-well plate at 100 μL/well. Bacteria were prepared in MHB broth with OD_630_ = 0.02 (final OD = 0.01, bacteria 10^6–7^ CFU/mL). Then, bacteria were transferred to a 96-well plate that contained AgNPs in equal volumes. The plates were incubated at 37 °C for 24 h in an incubator. After incubation, a clear solution was brought to 50 μL for a serial 10-fold dilution plate count with sterile phosphate-buffered saline (PBS) in triplicate. Then, 10 μL of each dilution was dropped on MH agar and cultured at 37 °C for 24 h to count the colony forming units per millilitre. A sterile PBS buffer was used as a no-treatment control. Gentamicin was used as positive control. The MIC value is the lowest concentration of agent that inhibits 90% of bacterial growth, and the MBC value is the lowest concentration of agent that inhibits 100% of bacterial growth. The percent inhibition was calculated using the following formula: [1 − (CFU sample/CFU control)] × 100 [26]. All measurements of the MIC and MBC values were repeated in triplicate for three independent experiments.

### 2.5. Staphylococcus pseudintermedius Cell Morphological Change

To observe the morphology of bacteria after treatment with AgNPs, field emission scanning electron microscopy (FESEM) and focused ion beam (FIB) or FIB-FESEM, a high-resolution technique, were used to investigate the morphological changes in cells after agent treatment. A single colony of *S. pseudintermedius* MIC411 was grown in MHB broth at 37 °C for 18–20 h. The bacteria were then inoculated with the same broth solution for 3 h. The bacteria were harvested by centrifugation twice in sterile deionised water. The incubation of cells (10^6^ CFU/mL) with gentamicin or AgNSs or anisotropic AgNPs was conducted at 37 °C for 1.5 h. The cells without treated agents served as the control. The bacterial cells were washed in PBS three times and dropped on an SEM membrane. The cells were fixed in 2.5% glutaraldehyde for 1 h and dehydrated with an ethanol series (30%, 50%, 70% and 90%) for 10 min, followed by 100% ethanol three times. After drying, the samples were observed under FIB-FESEM [27].

### 2.6. Cytotoxicity Assay

The cytotoxicity of AgNPs was evaluated by the 3-(4,5-dimethylthiazolyl-2)-2,5 diphenyltetrazolium bromide (MTT, AppliChem GmbH, Darmstadt, Germany) assay, the cell proliferation rate, and the reduction in cell viability when metabolic events led to apoptosis or necrosis. MTT, which is a yellow compound, is reduced by mitochondrial dehydrogenases into an insoluble purple formazan in living cells. A solubilization agent, such as dimethyl sulfoxide, is added to dissolve the formazan crystal. The colour can be measured in the range of 500–600 nm by a spectrophotometer [28].

HaCaT cells and NHDF cells were used to evaluate the cytotoxicity of AgNPs in different types of human cells. The cells were prepared in a 96-well plate at a concentration of 1 × 10^4^ and 8 × 10^3^ cells/well for the HaCaT and NHDF cells, respectively. After 24 h, AgNPs at different concentrations (0–100 μg/mL) were added to the plate. Cells without treated agents served as the control. To evaluate their viability, the cells were incubated and examined after 24 h. After this period of treatment, 10 μL of 5 μg/mL MTT stock solution was added to each well and incubated for 4 h at 37 °C. The medium was removed, and 150 μL of dimethyl sulfoxide (DMSO) was added into each well to dissolve the formazan crystals. The solution was measured at 570 nm by a Varioskan™ LUX multimode microplate reader (Thermo Scientific, Waltham, MA, USA). Experiments were performed in triplicate. Cell viability was presented as a bar graph between the percentage of cell viability (Y-axis) and the concentrations of AgNPs (X-axis), and a concentration of 50% cytotoxicity (IC_50_) was calculated. Cell viability was calculated using the following formula:

$$\mathrm{Cell}\;\mathrm{viability}\;\left(\%\right)\;=\frac{\mathrm{Absorbance}\;\mathrm{of}\;\mathrm{treated}\;\mathrm{cell}\;}{\mathrm{Absorbance}\;\mathrm{of}\;\mathrm{control}\;\mathrm{cell}}\;\times \;100$$

The IC_50_ value was calculated from a line graph of cell viability. It was defined as a 50% reduction of the absorbance or 50% of the percentage of cell viability [29].

### 2.7. Preparation and Formulation of the AgNPs Gel

To use AgNPs in the form of an antimicrobial gel, poly(acrylic acid) (Sigma-Aldrich, St. Louis, MO, USA) was used to prepare the gel. Briefly, poly(acrylic acid) was dissolved in sterile deionized water and neutralized by triethanolamine (Sigma-Aldrich, St. Louis, MO, USA). AgNPs (S1, S2 and S3) were added to the gel containing final AgNPs concentrations of 40 µg/g gel. The AgNPs gel was stored at room temperature until use. 

### 2.8. Antimicrobial Test and the Prolonged Antimicrobial Effect of the AgNP Gel

The antimicrobial efficiency of the AgNPs gel was determined by the agar well diffusion method. Bacteria were grown in MHB broth at 37 °C for 24 h. After inoculation for 3 h, the bacteria were diluted in the same media at an inoculum of 1 × 10^7^ CFU/mL. Then, the bacteria were swabbed onto a three-dimensional MH agar plate. A hole with a diameter of 6 mm was punched aseptically with a sterile cork borer. The AgNPs gel was filled into the wells at 200 µL and incubated at 37°C for 24 h. Povidone iodine (Leopard medical brand Co., Ltd., Nakorn Pathom, Thailand) was used as the positive control, and the gel without AgNPs as the negative control. After 24 h of incubation, the inhibition zone was measured on the millimetre (mm) scale. For a prolonged antimicrobial effect of the AgNPs gel, a test was performed with *E. coli*, *S. aureus*, *P. aeruginosa* and five isolates of *S. pseudintermedius*. The plates were incubated continuously in the same incubator for 48 h, and the observed inhibition zone was compared for 24 h [30].

### 2.9. Statistical Analysis

All data were expressed as the mean ± standard deviation (SD). The Student’s *t*-test was performed to analyze the significance of differences between the treated group and the control group (without AgNPs exposure). Analysis of variance with Tukey’s test was used for multiple comparisons. A *p*-value of less than 0.05 was considered significant.

## 3. Results

### 3.1. Characterisation of AgNPs

**Figure 1:**
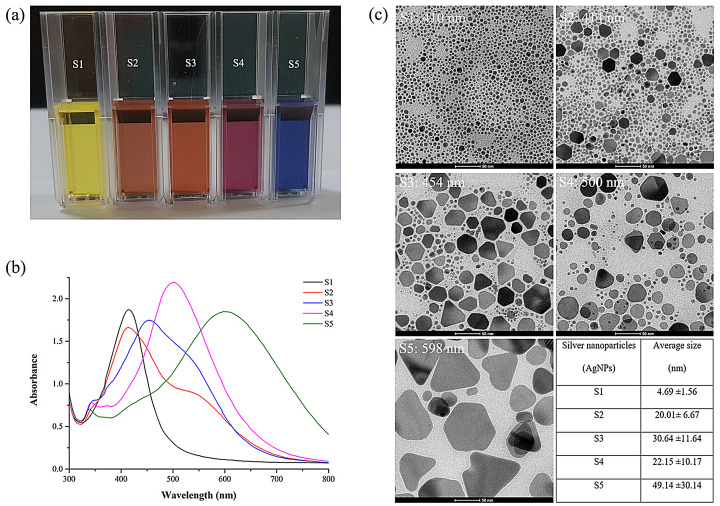
Characterization of AgNPs produced by chemical synthesis. (**a**) AgNP solution (S1–S5), (**b**) UV-Vis absorption spectrum of solutions containing AgNPs and (**c**) TEM image of AgNPs (scale 50 nm). The table for particle size distribution was produced using Image J software.

To evaluate the characteristics of AgNPs, UV-Vis spectroscopy was used to investigate the formation of AgNPs by assessing the signature surface plasmon resonance (SPR) bands. These optical features can be used to assess the shape, size and distribution of the nanostructures in the solution. We also monitored and confirmed the shape and size of AgNPs by TEM. A digital photograph of AgNPs (S1–S5) exhibited different vivid colours in the following order: pale yellow, dark-orange, light-orange, purple red and blue when viewed with the naked eye (Figure 1a). AgNSs (S1) appeared in yellow with a clear absorbance peak at 410 nm, showing the presence of lone spherical Ag nanoparticles (Figure 1b), which were confirmed by TEM imaging to have an average size of 4.69 nm ± 1.56 nm (Figure 1c). Anisotropic AgNPs (S2–S5) showed absorbance broad peaks at λ_max_ of 414, 454, 500 and 598 nm, respectively. Additionally, small peaks were found in the absorption spectra of S2–S5 in the range of 320–360 nm, and low broad peaks were found for S2 and S3 at 500–600 nm. Corresponding to the absorption spectra, the TEM image showed the shape of S2–S5 with a mixture of nanospheres and nanoplates (i.e., circular disks, truncated triangles and hexagons), and the amount of those with a spherical shape decreased from S2 to S5. From the TEM image, we calculated the size of AgNPs using Image J software (the table in Figure 1c). The average size of S1–S5 tended to increase with an average size of 4.69 ± 1.56, 20.01 ± 6.67, 30.64 ± 11.64, 22.15 ± 10.17 and 49.14 ± 30.14 nm, respectively. 

### 3.2. Bacterial Susceptibility to Antimicrobial Agents

The MIC is the lowest concentration of AgNPs that inhibits the growth of bacteria after overnight incubation. The bacteria used in this experiment are displayed in Table 1. The MIC values of S1–S5 of AgNPs against *E. coli* O157:H7, *S. aureus* ATCC 25923 and five isolates of *S. pseudintermedius* were found to be 1–16, 2–32, 2–100, 4–64 and 8–100 μg/mL, respectively (Table 2). The MBC is the lowest concentration of AgNPs required to kill bacteria after overnight incubation. The MBC values of S1–S5 AgNPs against the bacteria were found in the range of 2–32, 2–32, 2–100, 8–100 and 16–100 μg/mL, respectively (Table 2). In addition, the MIC and MBC values of gentamicin were in the range of 0.25–32 μg/mL and 0.5–32 μg/mL, respectively. All bacteria were susceptible to gentamicin but *S. pseudintermedius* MIC 407 and MIC 411 were completely resistant to gentamicin. According to a previous report, the *S. pseudintermedius* antibiotic breakpoint used for *in vitro* susceptibility testing of gentamicin was 8 μg/mL [31]. These results show that AgNPs could inhibit growth and kill all tested bacterial pathogens. The antimicrobial effect of AgNPs tended to reduce the antimicrobial efficiency from S1 to S5. Among all AgNPs, S1 and S2 had the highest antimicrobial activity and were close to antimicrobial efficacy of gentamicin. Furthermore, the antimicrobial activity of all AgNPs against *E. coli* (Gram-negative bacteria) was the highest compared with that of other bacteria. For the animal pathogens, the resistant bacterial isolates, including *S. pseudintermedius* MIC 407, 408 and 411, the MIC and MBC values were slightly higher than the susceptible isolates. 

### 3.3. Morphological Changes in Bacterial Cells 

**Figure 2:**
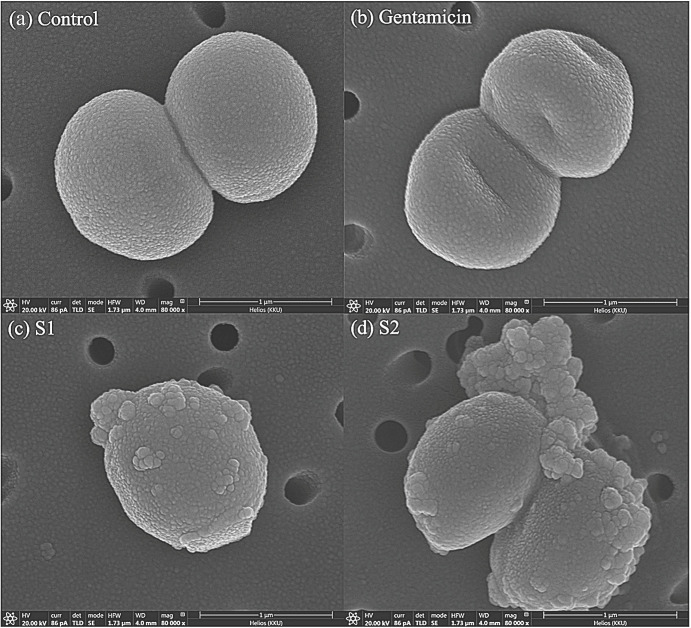
Cell morphological change of *S. pseudintermedius* MIC 411 observed by FIB-FESEM. Control cell (**a**). The bacterial cells were treated at concentration of MBC level for 1.5 h with gentamicin (**b**), AgNSs (**c**), and anisotropic AgNPs (**d**).Bacterial cell treated AgNPs (**c**,**d**) show the distorted cell, membrane blebbing, membrane damage, and clumping around the cell.

We observed morphological changes in bacterial cells to compare cell morphology after treatment with AgNPs. This study is the first to report on the cell alteration of *S. pseudintermedius* using FIB-FESEM. *S. pseudintermedius* MIC 411 was treated with AgNPs (S1 and S2) or gentamicin at a concentration of MBC for 1.5 h. As shown in Figure 2, the control cells, which were untreated, exhibited spherical cell integrity with smooth and damage-free cells (Figure 2a). The treated cells, which were treated with gentamicin, indicated collapse and distortion of the cells, as shown in Figure 2b. In the cells treated by AgNPs, including S1 and S2 (Figure 2c,d), the cells received aggressive damage, as exhibited by the rough outer cell wall and cell debris around the cell within 1.5 h. In addition, the bacterial cells treated with AgNPs revealed their distorted cell and membrane damage with disintegration and pores (Supplementary Figure S1). The results confirm extensive damage to the cell membrane in the presence of AgNPs compared with gentamicin and untreated cells.

### 3.4. Cytotoxicity Assay

**Figure 3:**
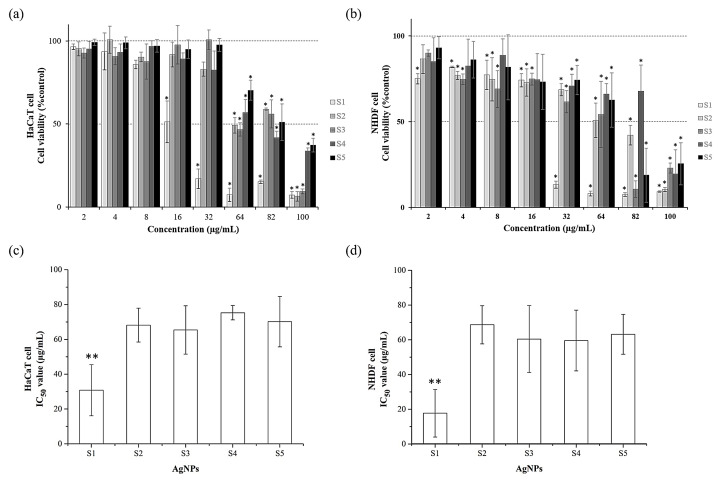
Cytotoxicity of AgNPs evaluated by MTT assay. Cell viability of HaCaT and NHDF cells (**a**,**b**) and IC_50_ of AgNPs in HaCaT and NHDF cells (**c**,**d**). The cells were incubated with five types of AgNPs (S1–S5) at 2–100 µg/mL for 24 h. The cell without AgNPs was the control with 100% viability. Data represent mean value ± SD (error bar) from two independent experiments carried out in triplicate (*n* = 6). An asterisk (*) indicates significant differences in comparison to the control without AgNPs (*p* < 0.05). ** Asterisks denote a significantly different *p*-value (*p* < 0.05) comparing AgNSs with other groups.

To examine the cytotoxic effect of AgNPs on human cells, the mitochondrial metabolic activities (MTT assay) of the HaCaT and NHDF cells were determined. The viability of the cells in the medium containing 2–100 μg/mL of AgNPs (S1–S5) was assessed after exposure for 24 h. The results show that AgNPs had cytotoxic effects in a dose-dependent manner (Figure 3). As shown in the bar graph, cell viability was clearly different between AgNSs (S1) and anisotropic AgNPs (S2–S5) in both keratinocytes and fibroblasts (Figure 3a, b). For the HaCaT cell, there was a statistically significant effect on cell viability in the presence of 16 μg/mL for AgNSs and 64 μg/mL for all anisotropic AgNPs compared to untreated control (Figure 3a). For the NHDF, there was a statistically significant effect on cell viability in the presence of 2 μg/mL for AgNSs, 4 μg/mL for S2–S3, and 32 μg/mL for S4–S5 compared to untreated control (Figure 3b). All AgNPs showed a lower cell viability of 50% at 100 μg/mL. The results also indicate that S1 had an IC_50_ value significantly lower than S2–S5 (*p* < 0.05). As shown in Figure 3c, the 50% cell viability of S1–S5 in HaCaT cells was shown to be 30.80 ± 14.67, 68.20 ± 9.71, 65.39 ± 13.84, 75.27 ± 4.11 and 70.17 ± 14.49 μg/mL, respectively. Similarly, S1–S5 showed a 50% cell viability in NHDF cells at concentrations of 17.70 ± 13.67, 68.65 ± 10.97, 60.42 ± 19.24, 59.58 ± 17.44 and 63.17 ± 11.51 μg/mL, respectively (Figure 3d). The results show that AgNSs (S1) had a 2–4-fold higher toxic effect than anisotropic AgNPs (S2) and anisotropic AgNPs showed moderate cytotoxicity to human cells. 

### 3.5. Antimicrobial Test of the AgNP Gel and Its Prolonged Antimicrobial Effect

**Figure 4:**
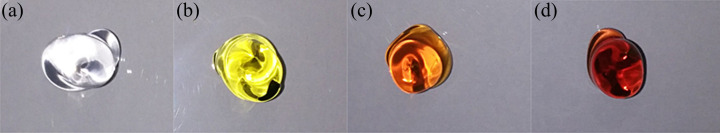
AgNPs containing formulation (AgNPs gels). (**a**) Control gel (without AgNPs), (**b**) AgNPs gels of S1, (**c**) S2 and (**d**) S3.

**Figure 5:**
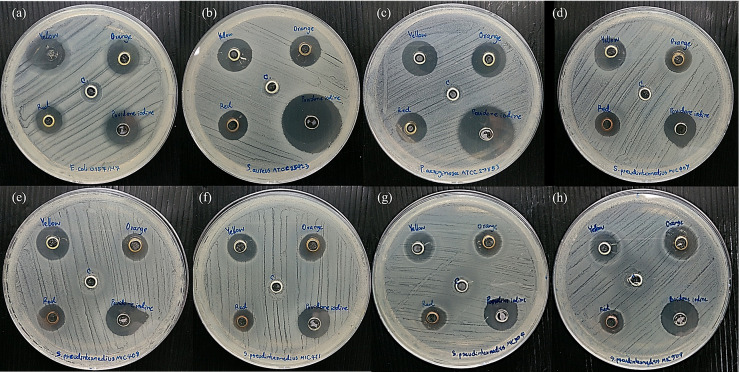
Antimicrobial activity of the AgNP gel determined by the well diffusion method. The AgNP gel (S1 [yellow], S2 [orange], S3 [red]), povidone iodine and control gel were dropped into wells and incubated with pathogens for 24 h at 37 °C. The inhibition zones were measured in mm in diameter. (**a**) *E. coli*, (**b**) *S. aureus*, (**c**) *P. aeruginosa*, (**d**–**h**) *S. pseudintermedius* MIC 407, 408, 411, 504, 509, respectively.

After determining the antimicrobial and cytotoxic effects, we producedan alternative form to use AgNPs, a gel formulation containing AgNPs for topical use. The gel was formulated from poly(acrylic acid), which was added with AgNPs at an optimal concentration. AgNSs (S1) and anisotropic AgNPs (S2 and S3) were used because they exhibited a good antimicrobial effect. A diffusion test was performed to determine the antimicrobial activity of the AgNP gel. We compared the antimicrobial activity of AgNPs gel with that of povidone iodine, which is an antiseptic for the prevention of wound infection in animals. The AgNP gel (Figure 4) had a vivid colour and suitable viscosity. As shown in the diffusion test results, all AgNP gels (S1–S3) displayed an inhibition zone on the bacterial agar plate (Figure 5). The average size of the inhibition zone of the AgNP gel (S1–S3) against the bacteria was 11.46 ± 0.99, 10.85 ± 0.46 and 9.53 ± 0.31 mm, respectively (Supplementary Table S1). The average inhibition zone of povidone iodine was 15.36 ± 1.04 mm, and no inhibition zone was found in the gel with no AgNPs. Based on the results, the inhibition zone of povidone iodine was significantly larger than that of S1, S2 and S3 (*p*-value < 0.05). In the comparison among the groups of AgNPs, the average sizes of the inhibition zones of S1, S2 and S3 were not significant. 

**Figure 6:**
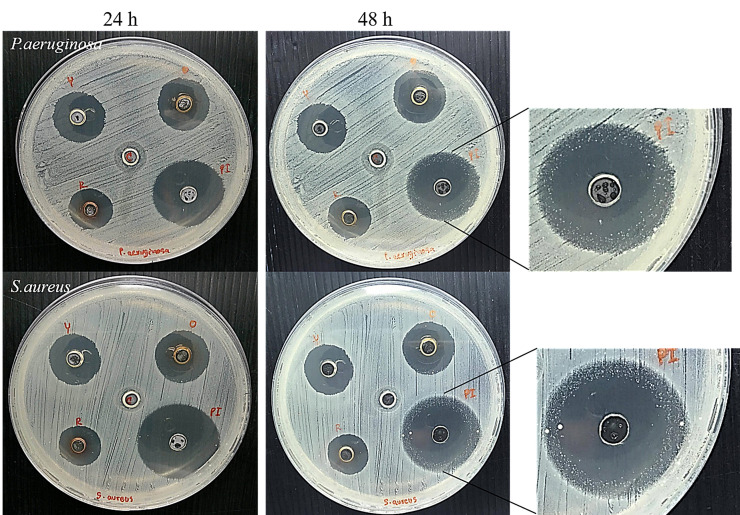
Antimicrobial activity of the AgNPs gel determined by the well diffusion method. The AgNP gels (S1 [yellow], S2 [orange], S3 [red]), povidone iodine and control gel were dropped into wells and incubated with pathogens for 48 h at 37 °C. The inhibition zones were measured in mm in diameter at 24 h and 48 h.

The long-term activity of the antimicrobial agent causes the reduced frequency of using antimicrobial agents. A prolonged antimicrobial effect test was performed on *E. coli*, *S. aureus*, *P. aeruginosa* and five isolates of *S. pseudintermedius*. After the agents were incubated with bacteria for 48 h, the inhibition zone of the AgNP gel remained the same at 24 h, whereas povidone iodine showed a decreased inhibition zone against *S. aureus* and *P. aeruginosa* (Figure 6). The bacteria could encroach the inhibition zone, thus decreasing the inhibition zone width from 22.50 mm ± 4.95 mm to 17.00 mm ± 2.65 mm and from 22.50 mm ± 0.71 mm to 15.00 mm ± 0.00 mm for *S. aureus* and *P. aeruginosa*, respectively (Supplementary Table S2). This indicates that the antimicrobial activity of povidone iodine was reduced after 48 h, whereas that of AgNPs remained active.

## 4. Discussion

As the resistance of bacteria to antibiotics is increasing in both human and animal pathogens, AgNPs are considered a new material for treating these pathogens. They are effective in combating antibiotic-resistant pathogens. The biological activity of AgNPs depends on size and shape. Our goal was to develop an antimicrobial gel formulation containing AgNPs, which could be an alternative to conventional antimicrobial agents for topical use. In this study, AgNSs and anisotropic AgNPs of different shapes and sizes were investigated for their efficacy against several bacterial pathogens and their cytotoxicity.

AgNPs were obtained from our collaborative company (Prime Nanotechnology Co., Ltd. Bangkok, Thailand). The characteristics of AgNPs obtained using UV-Vis spectrophotometry and TEM showed that AgNPs had different sizes, shapes and solution colours. AgNSs are spherical and usually show a single absorbance peak with absorption of about 400 nm [32]. Conversely, anisotropic AgNPs (S2–S5) exhibited more than one peak, indicating the presence of nanoparticles with different shapes. Moreover, they could shift to longer wavelengths with increasing particle size [33], as confirmed by TEM imaging. The absorption peaks were in the range of 320–360 nm and 500–600 nm for anisotropic AgNPs (S2–S5), implying the presence of triangular, hexagonal and truncated triangular nanoplates, which is similar to the results reported by Tewarak Parnklang et al., 2013 and 2015 [34,35]. Moreover, the red shift of anisotropic AgNPs indicates an increase in particle size from S2 to S5. Smaller nanoparticles showed absorption maxima at shorter wavelengths, whereas red shifts in maxima occurred when the particle size increased [36]. Clearly, AgNSs with a small spherical size showed only a single narrow absorption peak, whereas anisotropic AgNPs could exhibit two or more absorption bands depending on the shape of the particles.

Studies have investigated the antibacterial efficiency of anisotropic AgNPs (e.g., triangular prisms, hexagons, truncated triangles, etc.) and found that they had a higher antimicrobial properties than AgNSs [23,24]. The antimicrobial activity of AgNSs and anisotropic AgNPs was evaluated depending on the physical properties, especially size and shape [37,38]. After characterization, we estimated the antimicrobial activity of AgNPs against human and animal pathogens using the MIC and MBC values. The results show that the antimicrobial efficiency was followed by S1, S2, S3, S4 and S5, which can be divided into two groups: S1–S2 (MIC/MBC ≤ 32 µg/mL) and S3–S5 (MIC/MBC ≤ 100 µg/mL). Our results are the same as a previous report by Gabriele Meroni et al., 2020 and Katarzyna A et al., 2017 [39,40]; AgNPs have strong antibacterial abilities to *S. pseudintermedius*. In a comparison of all AgNPs, S1 and S2 were very close in MIC and MBC. Note that the antimicrobial activity of AgNPs had a tendency to decrease when particles transformed into anisotropic particles. The results also imply that the increment of anisotropic shape did not increase antimicrobial activity. The reason is that due to the transformation of AgNSs into anisotropic AgNPs resulting in an increased size, size greatly affects the antimicrobial property, with a larger size indicating a lower antimicrobial activity as shown in a previous report by Zhong Lu et al., 2013 [41]. A smaller size is able to enter the cell, whereas a larger size may pass slightly or not pass through into the cytosol [42]. Moreover, the smaller size could release silver ions more than the larger size, which would affect antimicrobial activity [43]. For these reasons, anisotropic AgNPs (S2) consisting of small spherical particles and smaller anisotropic shapes show an antimicrobial activity close to that of AgNSs. Conversely, other anisotropic AgNPs (S3–S5) with a larger size and consisting of few small spherical particles had lower antimicrobial activity. In addition, the highest antimicrobial activity of all AgNPs was found against *E. coli* because a Gram-negative bacteria has a membrane much thinner than a Gram-positive bacteria that could act as a protective layer [44]. The antimicrobial activity of AgNPs against MRSP was slightly lower than that against MSSP. This could be due to some cell components, or a resistance mechanism not found in MSSP. This issue should be examined in future research to efficiently manipulate MRSP using AgNPs.

We observed the cell morphology to study the effects of AgNPs that cause cell death. The results show that the morphology of bacterial cells was completely different after treatment with gentamicin and AgNPs. Gentamicin, which is an aminoglycoside antibiotic, acts by irreversibly binding to the 30s subunit of the bacterial ribosome and interfering with protein synthesis [45]. Thus, the bacterial cell collapsed and distorted because of the detrimental effect on the integrity of the cell wall. Conversely, cells treated with AgNPs (S1 and S2) showed quick and severe cell damage (within 1.5 h), as indicated by the rough outer cell wall and cell debris around the cell. Many blebs also appeared on the bacterial surface, implying the loss of cell membrane integrity by cell burst. Our result was the same as previously reported by Reham Samir Hamida et al., 2020; bacteria treated with AgNPs exhibited an apoptosis-like response in MRSA [46]. We speculated that this might be caused by the generation of reactive oxygen species (ROS). As the mechanism of action of AgNPs is unclear at present, it is possible that bacterial cells may produce many ROS molecules, such as superoxide anions, hydrogen peroxide and hydroxyl radicals [47]. ROS induction is considered to have an effect on several steps of the apoptosis cascade. ROS can also induce a bacterial apoptosis-like response by causing damage to cellular components [48]. These results show that the morphological change by treatment with AgNSs and anisotropic AgNPs is not different when observed under a microscope. We assume that the mechanisms of action of both AgNSs and AgNPs could be similar. AgNSs (S1) and anisotropic AgNPs (S2), which have a smaller size with a high surface area that could release Ag ions faster, could provide better contact with the microorganisms by binding to the cell membrane and penetrating inside [49,50], resulting in the antimicrobial activity of S1 and S2 being greater than that of other anisotropic AgNPs. Similar to proposed mechanism of action of AgNPs in the previous review, small particles can penetrate inside cells resulting in disruption of the bacterial envelope. Moreover, the particles and ions in a cell can bind to DNA and protein, including the induction of ROS production. These processes denature the cell membrane and rupture organelles, and even result in cell lysis [51]. However, the mechanisms of action of AgNPs should be studied more in the future to confirm the action on cells.

Aside from antibacterial efficacy, cytotoxicity to mammalian cells is also important to the development of antimicrobial agents. AgNSs are very toxic, whereas anisotropic AgNPs are moderately cytotoxic when compared to untreated control. Importantly, the antimicrobial effects of AgNSs and anisotropic AgNPs (S2) are not different (MIC [S1] 1–16 µg/mL and [S2] 2–32 µg/mL; equal MBC 2–32 µg/mL). Note that anisotropic AgNPs showed lower cytotoxicity than AgNSs by 2–4-fold (compared to IC_50_). Our results are the same as previously studied by Priscila L.L. Freire et al., 2016; AgNSs have shown more cytotoxicity than anisotropic AgNPs against murine macrophages [52]. AgNSs exhibited toxic effects that can be explained by the smaller nanoparticle sizes. There was a previous study that exhibited that AgNSs of 5 nm have high cytotoxicity when compared to others (25 nm, 50 nm, 110 nm) [53]. Thus, smaller AgNSs showed more toxicity to mammalian cells. In exhibiting their antimicrobial activity, smaller AgNSs could pass through the bacterial cell membrane into the cytosol and damage the components in the cytosol, such as protein, DNA, lipids and organelles [48]. Another way is to release Ag ions from smaller particles faster than from large particles, as smaller particles have a greater surface-area-to-volume ratio than large particles, thus oxidising smaller particles more easily [54]. It is clear how AgNSs showed more cytotoxicity than anisotropic AgNPs. In this case, the surface-area-to-volume ratio, that is, the rate that particles release Ag ions, is an important factor in the antimicrobial activity and cytotoxicity of AgNPs. AgNSs showed a more severe biological activity because AgNSs (S1) are smaller than anisotropic AgNPs. In contrast, Anisotropic AgNPs (S2) consist of small nanospheres and smaller anisotropic particles; they show both good antimicrobial action and low cytotoxic effect.

A good antimicrobial agent requires optimum properties, including good antimicrobial activity and low cytotoxicity. AgNPs at a concentration of the MBC level with low cytotoxicity to human cells were collected. The AgNP gel exhibited great antimicrobial activity in all bacteria. The average inhibition zone of the AgNP gel was smaller than that of povidone iodine because povidone iodine was in a solution, which shows fast action and diffuses more than the AgNP gel. Interestingly, the antimicrobial activity of AgNPs showed long-lasting protection against bacteria compared with povidone iodine. The remaining inhibition zone of the AgNP gel after 48 h could be caused by the action of Ag ions released from AgNPs [55,56]. Ag ions can be released in increments, depending on the oxidation of AgNPs by oxygen, antibacterial substances, chloride and peroxide. Our results are similar to those of previous studies. AgNPs have a longer activity than Ag ions and AgNO3 because AgNPs slowly release Ag ions, whereas AgNO_3_ has fast action in the entire Ag ion and disperses when neutralized [26,57]. AgNPsare a strong candidate for antimicrobial agent development because they have the advantage of long-time protection in animals that does not require frequent wound cleaning.

## 5. Conclusions

AgNPs have the potential to combat bacteria in infected animals and humans. The particle size and shape of AgNPs are key factors in their biological activity. As shown in this study, although the antibacterial activity of AgNSs (S1) was slightly higher than that of anisotropic AgNPs, it was also highly toxic to human cells. In summary, anisotropic AgNPs (S2) could be eligible for use as an alternative antibacterial agent because of their combined spherical and anisotropic shapes. The combination of size and shape resulted in good antimicrobial activity and reduced toxicity to human cells. In addition, the fast action of AgNPs to kill bacteria could be useful to reduce the chance that they might induce bacterial resistance. Therefore, anisotropic AgNPs (S2) might be a promising way to decrease the amount of antibiotic-resistant bacteria and an alternative antimicrobial agent for animals. Moreover, the proposed AgNPs gel showed prolonged antimicrobial efficiency, making it eligible as a treatment agent for infectious wounds in animals. This research also paves the way for future studies by revealing the optimal ratio of spherical and anisotropic AgNPs to fight bacteria and prolong antibacterial activity, and their low cytotoxicity to human cells for the effective use of anisotropic AgNPs.

## Figures and Tables

**Table 1:** Bacteria used in this study.

Bacteria	Sources
*E. coli* O157:H7	Human gastrointestinal tract
*S. aureus* ATCC 25923	Clinical isolate
*P. aeruginosa* ATCC 27853	Hospital blood specimen
* *S. pseudintermedius* MIC 407	Crust from skin
* *S. pseudintermedius* MIC 408	Papule/recurrent pyoderma
* *S. pseudintermedius* MIC 411	Exudate from wound
*S. pseudintermedius* MIC 504	Pustule
*S. pseudintermedius* MIC 509	Deep pyoderma

* Methicillin-resistant *Staphylococcus pseudintermedius* (MRSP).

**Table 2:** Minimum inhibitory concentration (MIC) and minimum bactericidal concentration (MBC) of AgNPs against bacteria by serial dilution plate count assay.

Bacteria	MIC (µg/mL)						MBC (µg/mL)					
	S1	S2	S3	S4	S5	GENT	S1	S2	S3	S4	S5	GENT
*E. coli* O157:H7	1–4	2–4	2–4	4–16	8–16	0.5	2–4	2–8	2–8	8–32	16–32	1
*S. aureus* ATCC 25923	8–16	32	16–32	16–64	32–100	1–2	16–32	32	32–64	100	100	2
* MIC 407	2–16	4–32	16–32	32–64	32–100	32	16	4–32	16–32	32–64	32–100	32
* MIC 408	4–16	4–32	8–64	32–64	64–100	2–4	4–16	4–32	16–64	32–100	64–100	2–4
* MIC 411	2–16	4–32	16–100	32–64	32–100	16–32	4–32	8–32	32–100	32–100	64–100	16–32
MIC 504	2–4	4–16	16–32	16–32	32–64	0.5	2–8	4–16	32	32–64	64	1–4
MIC 509	2–4	4–16	4–32	16–64	32–64	0.25	4–8	8–16	16–32	16–64	32	0.5
Range of MIC/MBC	1–16	2–32	2–100	4–64	8–100	0.25–32	2–32	2–32	2–100	8–100	16–100	0.5–32

The MIC value corresponds to the lowest concentration that inhibits ≥ 90% of bacterial growth. The MBC value corresponds to the lowest concentration that inhibits 100% of bacterial growth. Experiments were performed in triplicate of three independent experiments for each cell type. The five isolates of *S. pseudintermedius* are MIC 407, 408, 411, 504 and 509. * Methicillin-resistant *Staphylococcus pseudintermedius* (MRSP); S1–S5 = AgNPs, GENT = Gentamicin antibiotic.

## Data Availability

The data presented in this study are available upon request from the corresponding author.

## Supplementary Materials

The following are available online at https://www.mdpi.com/article/10.3390/vetsci8090177/s1, Table S1: Inhibition zone diameter of AgNP gel (S1–S3) and povidone iodine against two species of human pathogens and animal pathogen (five isolates of *S. pseudintermedius*), Table S2: Comparison of the inhibition zone diameter of AgNP gel (S1–S3) with povidone iodine after the antimicrobial agents were incubated with bacteria for 48 h. Figure S1: Cell morphological change of *S. pseudintermedius* MIC 411 observed by FIB-FESEM. The bacterial cells were treated at concentration of MBC level for 1.5 h with AgNSs (a), and anisotropic AgNPs (b). Bacterial cell treated AgNPs show the distorted cell and membrane damage with disintegration and pores.
