# Supplementary material for: A Biological Study of Anisotropic Silver Nanoparticles and Their Antimicrobial Application for Topical Use
Source: Vet Sci. 2021 Aug 31;8(9):177. doi: 10.3390/vetsci8090177 (PMC8471216; doi:10.3390/vetsci8090177)
Supplement: Supplementary file 1 [file vetsci-08-00177-s001.zip › vetsci-1347421-supplementary.pdf]

Supplementary data

Table S1

Inhibition zone diameter of AgNP gel (S1–S3) and povidone iodine (PI) against two species of human pathogens and animal pathogen (five isolates of *S. pseudintermedius*).

**Bacteria**	**Inhibition zone diameter (mm)[a]**			
	**S1**	**S2**	**S3**	**PI**
E. coli O157/H7	14.00 ± 2.83	11.50 ± 0.71	9.00 ± 0.00	12.00 ± 0.00
S. aureus ATCC 25923	14.00 ± 0.00	13.00 ± 0.00	12.00 ± 0.00	22.50 ± 4.95
P. aeruginosa ATCC 27853	13.50 ± 0.71	12.00 ± 0.00	11.00 ± 0.00	22.50 ± 0.71
* MIC407	10.75 ± 1.06	10.00 ± 0.00	8.25 ± 1.06	12.88 ± 1.24
* MIC408	11.50 ± 0.71	9.25 ± 1.06	9.88 ± 0.18	13.00 ± 0.00
* MIC411	9.50 ± 0.71	8.75 ± 0.71	7.63 ± 0.53	12.50 ± 0.71
MIC504	8.12 ± 0.88	9.00 ± 0.00	8.50 ± 0.71	13.50 ± 0.71
MIC509	9.25 ± 1.06	10.62 ± 1.24	8.00 ± 0.00	14.00 ± 0.00
Average	11.46 ± 0.99	10.85 ± 0.46	9.53±0.31	15.36 ± 1.04**

[a]Mean value ± SD, the mean of the trilicate samples. Asterisk (**) denotes a statistically significant difference compared with other groups ($p < 0.05$). The inhibition zone includes the diameter of the well (6 mm). The five isolates of *S. pseudintermedius* are MIC 407, 408, 411, 504 and 509.

* Methicillin-resistant *Staphylococcus pseudintermedius* (MRSP)

Table S2

Comparison of the inhibition zone diameter of AgNP gel (S1–S3) with povidone iodine (PI) after the antimicrobial agents were incubated with bacteria for 48 h.

**Agents**	**Inhibition zone (mm)**[a]			
	S. aureus **ATCC 25923**		***P. aeruginosa*** **ATCC 27853**	
	**24 h**	**48 h**	**24 h**	**48 h**
S1	14.00 ± 0.00	14.00 ± 0.00	15.25 ± 0.35	15.25 ± 0.35
S2	14.25 ± 1.77	14.25 ± 1.77	14.63 ± 1.11	14.63 ± 1.11
S3	11.50 ± 0.71	11.50 ± 0.71	12.83 ± 1.03	12.83 ± 1.03
PI	22.50 ± 4.95	17.00 ± 2.65	22.50 ± 0.71	15.00 ± 0.00

[a]Mean value ± SD, the mean of the triplicate samples. The inhibition zone includes the diameter of the well (6 mm).

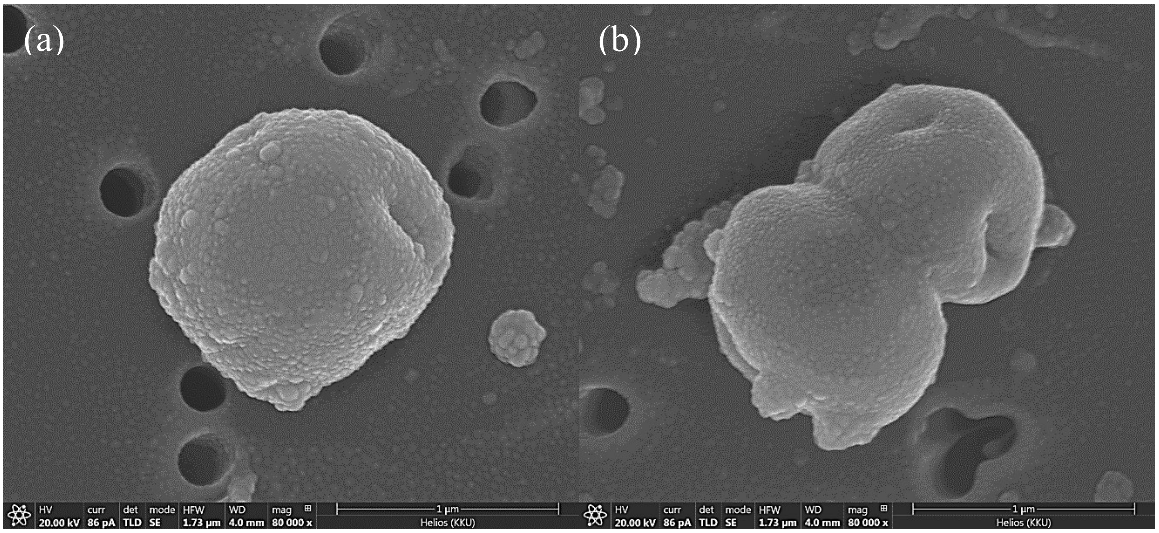

Figure S1 Cell morphological change of *S. pseudintermedius* MIC 411 observed by FIB-FESEM. The bacterial cells were treated at concentration of MBC level for 1.5 h with AgNSs (a), and anisotropic AgNPs (b). Bacterial cell treated AgNPs show the distorted cell and membrane damage with disintegration and pores.
